# Circulating T follicular helper 2 cells, T follicular regulatory cells and regulatory B cells are effective biomarkers for predicting the response to house dust mite sublingual immunotherapy in patients with allergic respiratory diseases

**DOI:** 10.3389/fimmu.2023.1284205

**Published:** 2023-11-24

**Authors:** Katsunori Shigehara, Ryuta Kamekura, Ippei Ikegami, Hiroshi Sakamoto, Masahiro Yanagi, Shiori Kamiya, Kentaro Kodama, Yuichiro Asai, Satsuki Miyajima, Hirotaka Nishikiori, Eiji Uno, Keisuke Yamamoto, Kenichi Takano, Hirofumi Chiba, Hirofumi Ohnishi, Shingo Ichimiya

**Affiliations:** ^1^ Department of Human Immunology, Research Institute for Frontier Medicine, Sapporo Medical University School of Medicine, Sapporo, Japan; ^2^ Department of Respiratory Medicine and Allergology, Sapporo Medical University School of Medicine, Sapporo, Japan; ^3^ Ai Medical Clinic, Sapporo, Japan; ^4^ Department of Otolaryngology and Head and Neck Surgery, Sapporo Medical University School of Medicine, Sapporo, Japan; ^5^ Department of Dermatology, Sapporo Medical University School of Medicine, Sapporo, Japan; ^6^ Department of Public Health, Sapporo Medical University School of Medicine, Sapporo, Japan

**Keywords:** house dust mite SLIT, T follicular helper cells, T follicular regulatory cells, B regulatory cells, Der-p/f-specific Igs

## Abstract

The relationships between T follicular helper (Tfh) cells and antigen-specific immunoglobulins (sIgs) in patients with allergic respiratory diseases who are receiving antigen immunotherapy (AIT) have not been fully clarified. Therefore, we started to perform house dust mite sublingual immunotherapy (HDM-SLIT) for 20 patients with atopic asthma comorbid with allergic rhinitis (AA+AR) who were already receiving ordinary treatments including inhaled corticosteroid (ICS). We examined percentages of circulating T follicular helper (cTfh) and regulatory (cTfr) cells and percentages of circulating regulatory T (cTreg) and B (cBreg) cells by FACS and we examined levels of *Der-p/f* sIgs by ELISA. Based on the symptom score (asthma control questionnaire: ACQ) and medication score ((global initiative for asthma: GINA) treatment step score) in patients with AA, the patients were divided into responders and non-responders. The percentage of cTfh2 cells significantly decreased and the percentage of cTfh1 cells significantly increased within the first year. *Der-p/f* sIgEs decreased after a transient elevation at 3 months in both groups. Notably, the percentage of cTfh2 cells and the ratio of cTfh2/cBreg cells and *Der-p/f* sIgEs greatly decreased in responders from 6 months to 12 months. The percentages of cTfr and cTreg cells showed significant negative correlations with the percentage of cTfh2 cells. The percentage of IL-4^+^ cTfh cells were significantly decreased and the percentage of IFN-γ^+^ cTfh cells were increased before treatment to 24 months in 6 patients examined (4 responders and 2 non-responders). We performed multi plelogistic regression analysis based on these results, the ratios of cTfh2/cTfr cells and cTfh2/cBreg cells at the start of therapy were statistically effective biomarkers for predicting the response to HDM-SLIT in patients with AA+AR.

## Introduction

Atopic asthma (AA) and allergic rhinitis (AR) are caused by an immunoglobulin (Ig) E-mediated hypersensitivity reaction to airborne allergens. Allergen immunotherapy (AIT) is the sole therapy that can improve the natural history of allergic diseases. It has been reported that AIT given continuously over a period of 3 years continued to have clinical benefits in more than three-quarters of AA and AR patients (1, 2). The patho-physiological mechanism of AIT is thought to be blocking of antigen-sIgG/IgG4 antibodies that can inhibit antigen-sIgE-dependent activation on mast cells and basophils. Suppression of Th2 immunity occurs as a consequence of either deletion or anergy of Th2 cells, caused by induction of regulatory T (Treg) cells and regulatory B (Breg) cells, and immune deviation in favor of a Th1 response (3, 4). A recent study on cases in which AIT was effective showed that T follicular regulatory (Tfr) cells, a specialized CD4^+^ T cell subset in follicles, are regulated by B-cell lymphoma (BCL) 6 and forkhead box P (Foxp) 3, which are shared by regulatory T (Treg) cells, and produce IL-10 and TGF-β and inhibit IgE-producing Tfh2 cells in allergic diseases (5). On the other hand, T follicular helper (Tfh) cells have been recognized as the central player in the regulation of B cells to support antibody production largely based on their localization in B cell follicles of germinal centers (6–8). It was shown that Tfh2 cells (a subset of circulating cTfh cells), but not Th2 cells, promote IgE production in an HDM-sensitized asthma model (9). Several studies including our study have indicated the possibility that Tfh2 cells participate in IgE production in AA and AR patients (10, 11). However, it remains unclear how antigen-specific Tfh cells control the production of antigen sIgE and sIgG4/IgG in patients with allergic diseases who are receiving AIT.

It has been well established that about two thirds of AA patients have AR, which is a serious risk factor for the development of severe asthma. Conversely, about one third of AR patients also have AA. It is clear that AR and AA have a common pathophysiological origin and they may be associated with environmental allergens and characteristic immunity. Medications used for treatment of AR appear to be useful for improving asthma control. Moreover, AIT not only reduces nasal and conjunctivitis symptoms but also improve the condition of asthma. Therefore, AA comorbid with AR (AA+AR) is an appropriate indication for AIT (12).

In this study, to further clarify the immunological relevance between Tfh cells and antigen sIgs in patients with AA+AR, the percentages of cTfh/cTfr cells including cTreg/cBreg cells were determined by FACS and levels of HDM-specific (s) IgE, IgG4 and IgG were measured by using ELISA. We found that there were several important immunological results and biomarkers among the cTfh/cTfr cells, cTreg/cBreg cells and HDM-sIgs in the course of AIT in patients with AA+AR.

## Methods

### Study population

Thirty-three patients with AA+AR were enrolled in this study. The characteristics of the patients are summarized (**Supplementary Table 1**). The diagnosis of AA + AR was based on Global Initiative for Asthma (GINA) 2022 (13), Japan Respiratory Society Guidelines (14) and ARIA 2016 (15). All of the patients had high levels of specific *Dermatophagoides pteronyssinus* (*Der-p*) and *farinae* (*Der-f*) IgEs and some specific IgEs to other allergens. Half of the AA + AR patients also had other allergic diseases including allergic conjunctivitis, atopic dermatitis and pollen food allergy syndrome. All of the subjects were non-smokers. Written informed consent was obtained from all of the subjects according to the Declaration of Helsinki. All of the protocols were approved by the Institutional Review Board of Sapporo Medical University Hospital in Japan.

### Study protocol

All of the patients had already been treated with inhaled corticosteroid (ICS) ± long-acting β2 agonist (LABA) inhalation and/or antihistamine, leukotriene receptor antagonist, nasal steroid spray or other drugs for internal use, and none of the patients had been receiving oral or intravenous drip of corticosteroids. ICS dosage was standardized to budesonide. All of the patients showed over 70 percentage of forced expiratory volume in one second (%FEV1.0) on the basis of GINA recommendation. In twenty patients, we administered a 10^4^U HDM-tablet (equal proportions of a 50%: 50% mixture of *Der-p* and *Der-f* (MITICURE^®^, Torii, Japan) equivalent to 6SQ-HDM). We examined symptom/medication scores (AA: asthma control questionnaire (ACQ) (16), GINA treatment step score (13) and AR: cSMS by European Academy of Allergy and Clinical Immunology (EAACI) (17)). The percentages of cTfh cells and their subsets cells, cTfr cells, cTreg cells and cBreg cells, were determined by FACS and serum titers of *Der-p* and *Der-f* IgE, IgG4, and IgG were measured by ELISA before administration of the tablet and at 3, 6, 12, 18, and 24 months after the start of administration. We defined responders to HDM-SLIT as patients who showed an improvement in ACQ and GINA treatment scores, and we defined non-responders to HDM-SLIT as patients who showed no change or worse scores during the 2-year period. ICS dose of each case was standardized to budesonide. Thirteen patients did not receive HDM-SLIT and only performed measurement of FACS and *Der-p/f* Igs at the start of study and 12 months.

### Antibodies

Panels of directly conjugated anti-human monoclonal antibodies were used to measure lymphocyte species of cTfh cells and their subset cells, cTfr cells, cTreg cells and cBreg cells, and intracellular cytokines (IL-4 and IFN-γ). The antibodies used are shown (**Supplementary Table 2**).

### Flow cytometry

Heparinized peripheral blood mononuclear cells (PBMCs) were isolated from fresh blood specimens by centrifugation over a discontinuous density gradient (Lympholyte-H; Cedarlane, Burlington, ON, Canada). Cell staining and flow cytometry were performed by using FACSCanto II (BD Biosciences, San Jose, CA, USA). All of the data were analyzed using FACSDiva software (BD Biosciences) and Flowjo Software (BD Biosciences) (18). We also examined expressions of intracellular IL-4 and IFN-γ using Cell Stimulation Cocktail pulse protein transport inhibitors^®^ and Intracellular Fixation & Permeabilization Buffer^®^ (Thermo Fisher Scientific, MA, USA) in a part of AA+AR patients. To detect secretory IL-10 of CD3^-^CD19^+^CD24^hi^CD27^+^ cells (Breg cells), we used IL-10 Secretion Assay Detection Kit (APC) ^®^ (Miltenyi Biotec B.V.&Co.KG, Bergisch Gladbach, Germany, http://www.miltenyibiotec.com) (19, 20).

### ELISA

We measured the serum levels of *Der-p* and *Der-f*-specific IgE, IgG4 and IgG by using the 3gAllergy-specific Igs assay (3gAllergy, Siemens Healthcare Diagnostic Inc.) (21).

### Statistical analysis

All data are shown as means ± SEM (**Supplementary Table 1** are shown as means± SD.). Significance in the data was determined by using Student’s t-test or Wilcoxson matched-paired signed rank test. Repeated one-way ANOVA or two-away ANOVA was used for analysis of data before and after continuing treatment. Two-way ANOVA was used to compare the pattern of change in variables over time between responders and non-responders (Tukey correction or Bonferroni correction were used between time points in same group and between two groups, respectively.), and an interaction term was used to determine differences in the pattern of change between two groups. Correlations were determined by Pearson’s correlation coefficient. In order to determine biomarkers for HDM-SLIT, multi plelogistic regression analysis with the backward variable selection method was performed for responders and non-responders as dependent variables. Probability values less than 0.05 were considered significant.

## Results

### *Responders* and non-responders based on ACQ, and GINA treatment steps scores and cSMS scores in patients with AA+AR who received HDM-SLIT

We investigated symptom and medication scores in patients with AA+AR who were treated by HDM-SLIT for two years. Fourteen patients showed improvement of ACQ and GINA treatment steps scores at 24 months compared with the scores at the start of HDM-SLIT (responders), but six patients showed no change in or worse ACQ and GINA treatment steps scores (non-responders). There were statistically significant interactions of ACQ and GINA treatment steps scores between the two groups and the scores decreased as time proceeded in responders. In non-responders, we could not decrease the ICS dose because of no change in or worse ACQs score. There were also significant interactions of cSMS between the two groups and these scores decreased as time proceeded in responders (**Supplementary Figure 1**).

The ICS dosage in responders at 24 months was 38.3% lower than that at the start of HDM-SLIT, but the ICS dosage in non-responders conversely increased. GINA treatment step score mainly means a reduction of ICS dose in asthmatic patients.

### Percentages of Tfh cells, Tfr, Treg and Breg cells and levels of Der-p/f Igs in responders and non-responders by HDM-SLIT in two years

**Figure 1:**
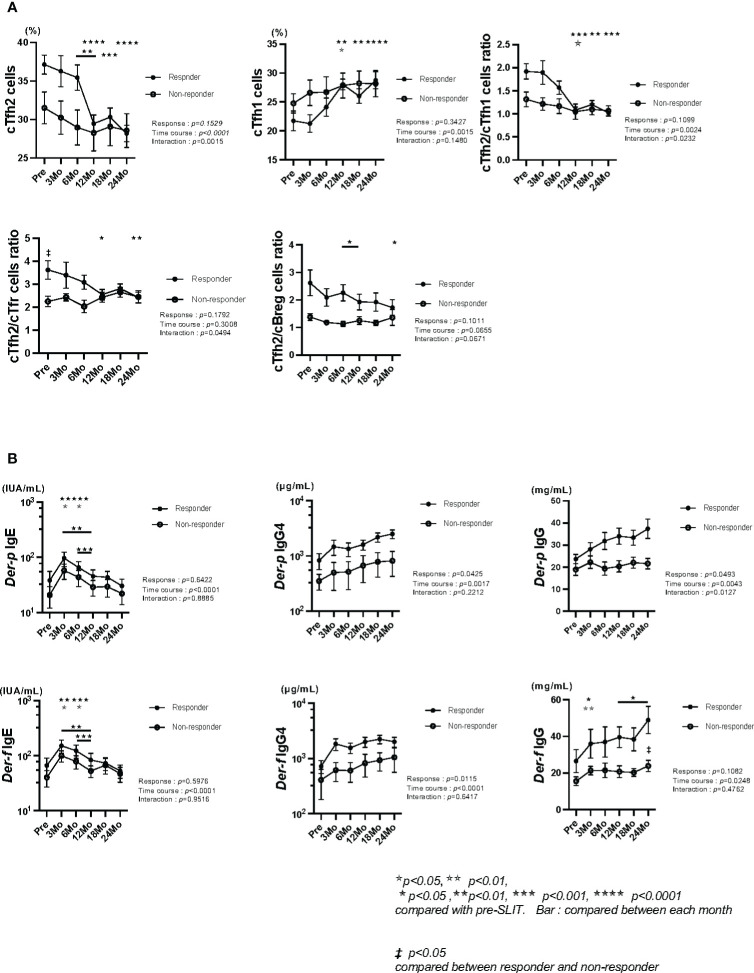
**(A)** Comparison of Tfh cell subsets, cTfh2/cTfr cells ratio and cTfh2/cBreg cells ratio between responders and non-responders. The percentage of cTfh2 cells and the ratio of cTfh2/cTfh1 cells showed significant interactions in responders and non-responders during the two-year period. The percentages of cTfh2 and cTfh1 cells significantly decreased and increased and the ratio of cTfh2/cTfh1 cells significantly decreased in responders. The ratio of cTfh2/cTfr cells showed a significant interaction and was significantly increased in responders compared with that in non-responders at the start of HDM-SLIT and significantly decreased at 12 and 24 months in responders. The ratio of cTfh2/cBreg cells was significantly decreased in responders from 6 months to 12 months and 24 months (***^‡^*** *p < 0.05 compared between the two groups, and ^★☆,^p<0.05*, *^★★^p<0.01, ^★★★^p<0.001, ^★★★★^p<0.0001 compared with pre-SLIT*). **(B)** Comparisons of *Der-p/f* specific-Igs between responders and non-responders. Levels of *Der-p/f* IgE significantly decreased from 6 months to 12 months. Levels of *Der-p/f* IgG4 showed a significant response in the responder group during the two-year period. Levels of *Der-p*IgG showed significant response, time course and interaction. Levels of *Der-f*IgG significantly increased in both groups and were significantly increased in responders compared with those in non-responders at 24 months (***^‡^*** *p < 0.05 compared between two the groups, and ^★,☆^p < 0.05*, *^★★,☆☆^p < 0.01*, *^★★★^p<0.001 compared with pre-SLIT*). ^★★★★★^, ^★★★★★★^ significance.

We examined the percentages of Tfh cells and their subsets, Tfr, Treg and Breg cells, and the levels of *Der-p/f* sIgs in responders and non-responders (FACS procedures in **Supplementary Figures 2**, **3**). The percentage of cTfh2 cells and the ratio of cTfh2/cTfh1 cells showed significant interactions and these significantly decreased from 6 or 3 months to 12 months in responders. The percentage of cTfh1 cells increased especially in responders (**Figure 1A**). (The absolute number of cTfh2 cells showed significant decreased in the first 12 months after the start of HDM-SLIT in responders (unpublished data)).

There were no significant differences between the two groups in responses, time courses and interactions in percentages of cTfr cells, cTreg cells and cBreg cells, but the percentage of cTreg cells in responders was significantly increased at 6 months compared with that before the start of HDM-SLIT (**Supplementary Figure 4**) and the percentage of cBreg cells significantly decreased from 3 months to 6 months (**Supplementary Figure 5**). The ratio of cTfh2/cTfr cells showed a significant interaction and this ratio in responders was significantly increased compared with that in non-responders at 6 months. The ratio of cTfh2/cBreg cells showed a significant decline from 6 month to 12 months and was significantly higher in responders than in non-responders before the start of HDM-SLIT (**Figure 1A**). The other cells at the start of HDM-SLIT to 24 months were described including absolute numbers (**Supplementary Figure 4**: cTfh subsets cells, cTreg cells and cTfr cells, **Supplementary Figure 5**: cB(total) cells, circulating (c)-memory B cells, c-plasmablast and cBreg cells).

We also measured the levels of *Der p/f*-sIgs. All of the levels of *Der p/f*-sIgs were higher in the responder group than in the non-responder group (**Figure 1B**). There were no significant differences in the levels of *Der p/f*-sIgEs between the two groups, and the levels were transiently increased at 3 months and gradually decreased thereafter. The levels of *Der-p/f* sIgG4s showed significant responses in the responders compared to those in the non-responders and showed significant time courses in both groups. The levels of *Der-p* sIgG showed a significant response, time course and interaction in responders. The levels of *Der-f* sIgG showed significant time course in responders.

When we observed the changes in cTfh2 cells and *Der-p/f* sIgEs during the 2-year period, there were no significant correlations between percentage of cTfh2 cells and levels of *Der-p/f* sIgEs (unpublished data), however, the percentage of cTfh2 cells and the levels of *Der p/f*-sIgEs remarkably decreased in all of the responders from 6 months to 12 months (cTfh2 cells: *p = 0.0012*, *Der-p* IgE: *p = 0.0004*, *Der-f* IgE: *p = 0.0001*). Although the levels of *Der p/f*-sIgEs also significantly decreased in the non-responders (*Der-p* IgE: *p = 0.0111*, *Der-f* IgE: *p = 0.0034*), there was no significant decrease in the percentage of cTfh2 cells in the non-responders. These results indicated a strong association between Tfh2 cells and *Der p/f*-sIgEs in the responder group. The ratio of cTfh2/cBreg cells also significantly decreased in the responders from 6 months to 12 months, but there was no significant change in the ratio in the non-responders (ratio of cTfh2/cBreg cells: responders, *p = 0.0114;* non-responders, *n.s.*) (**Figure 1A**).

### Relationship *between cTfh2 cells and regulatory cells in patients with AA+AR receiving HDM-SLIT*

**Figure 2:**
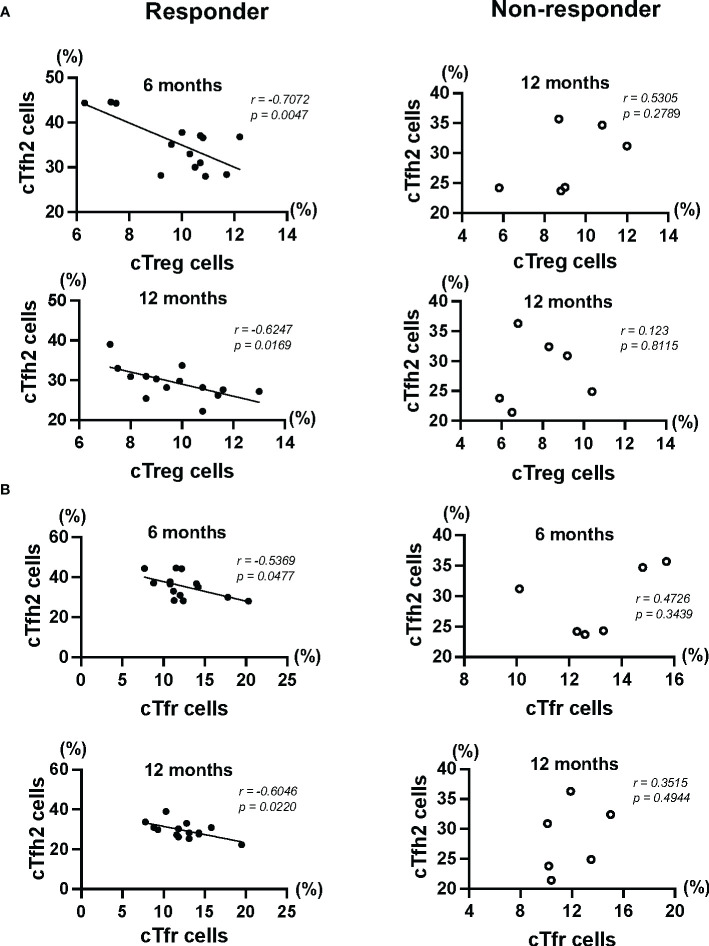
**(A)** The percentages of cTfh2 cells were significantly correlated with the percentages of cTreg cells (6 months: *r = - 0.7072*, *p = 0.0047*, and 12 months: *r = -0.6247*, *p = 0.0169*). **(B)** The percentages of cTfh2 cells showed strong significant correlations with the percentages of cPD-1^+^ Tfr cells (6 months: *r = -0.5369*, *p = 0.0048*, and 12 months: *r = -0.6064*, *p = 0.0220*).

Since the results showed the possibility of clear associations between the percentage of cTfh2 cells and levels of *Der p/f*-sIgEs from 6 months to 12 months in responders, we examined the relationship between the percentage of cTfh2 cells and the percentages of regulatory cells. The percentage of cTreg cells showed a significant negative correlation with the percentage cTfh2 cells at 6 months and 12 months in the responder group (**Figure 2A**). The percentage of cTfr cells also showed a strong negative correlation with the percentage of cTfh2 cells at 6 months and 12 months in the responder group (**Figure 2B**). However, there were no significant correlations between the percentages of these cells in the non-responders. It is speculated that suppression of Tfh2 cells by these regulatory cells results in reduction of *Der p/f*-sIgEs in HDM-SLIT responders.

### Percentages *of cTfh2 and cTfh1 cells with or without HDM-SLIT and expression of IL-4 and IFN-γ in cTfh cells with HDM-SLIT*

**Figure 3:**
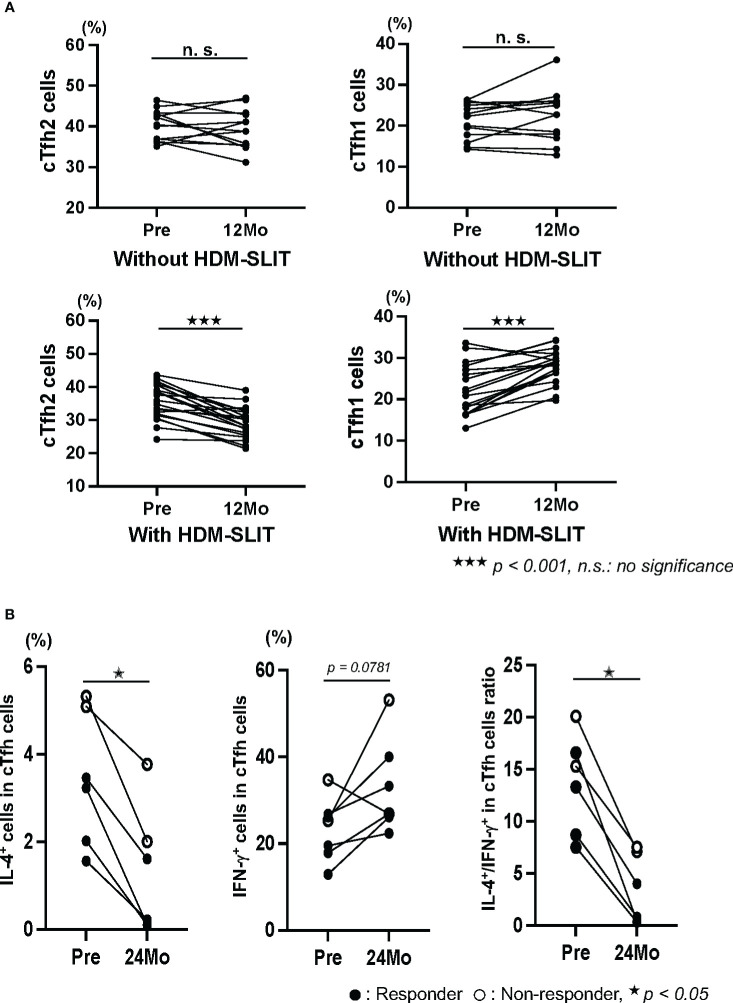
**(A)** The percentages of cTfh2 cells significantly decreased and the percentage of cTfh1 cells significantly increased in the first 12 months after the start of HDM-SLIT, but there were no changes in the percentages of those cells in patients who did not receive HDM-SLIT. Paired t-test was used (^★★★^ *p < 0.001*). **(B)** The percentage of IL-4^+^ cTfh cells significantly decreased and the percentage of IFN-γ^+^ cTfh cells increased between the start of HDM-SLIT and 24 months in 4 responders (cases 6,7, 8 and 13) and 2 non-responders (cases 16 and19). The ratios of IL-4^+^/IFN-γ^+^ cTfh cells were lower in the 4 responders than in the 2 non-responders. Wilcoxson matched-paired signed rank test was used (^★^ *p < 0.05*, ^★★^ *p < 0.01*). n.s., no significance.

Although the percentage of cTfh2 cells significantly decreased and the percentage of cTfh1 cells significantly increased in responders in the first year after the start of HDM-SLIT, there was no significant change in the percentages of cTfh2 and cTfh1 cells without HDM-SLIT during a period of one year (**Figure 3A**). We also examine that the intracellular expressions levels of IL-4 and IFN-γ in cTfh cells were measured by FACS in 6 patients (4 responders: cases 6, 7, 8, and 13 and 2 non-responders: cases 16 and 19) between the start of HDM and 24 months. The IL-4 expressions level significantly decreased. The IFN-γ expressions level did not significantly increase (*p = 0.0781*) in those patients, but the expression level increased in the patients except for one non-responder. The ratios of IL-4^+^/IFN-γ^+^ in cTfh cells significantly decreased (*p = 0.0321*, **Figure 3B**). The ratios in the 4 responders were lower than those in the 2 non-responders.

### Predictive biomarker of the response to HDM-SLIT based on findings at the start of therapy

**Figure 4:**
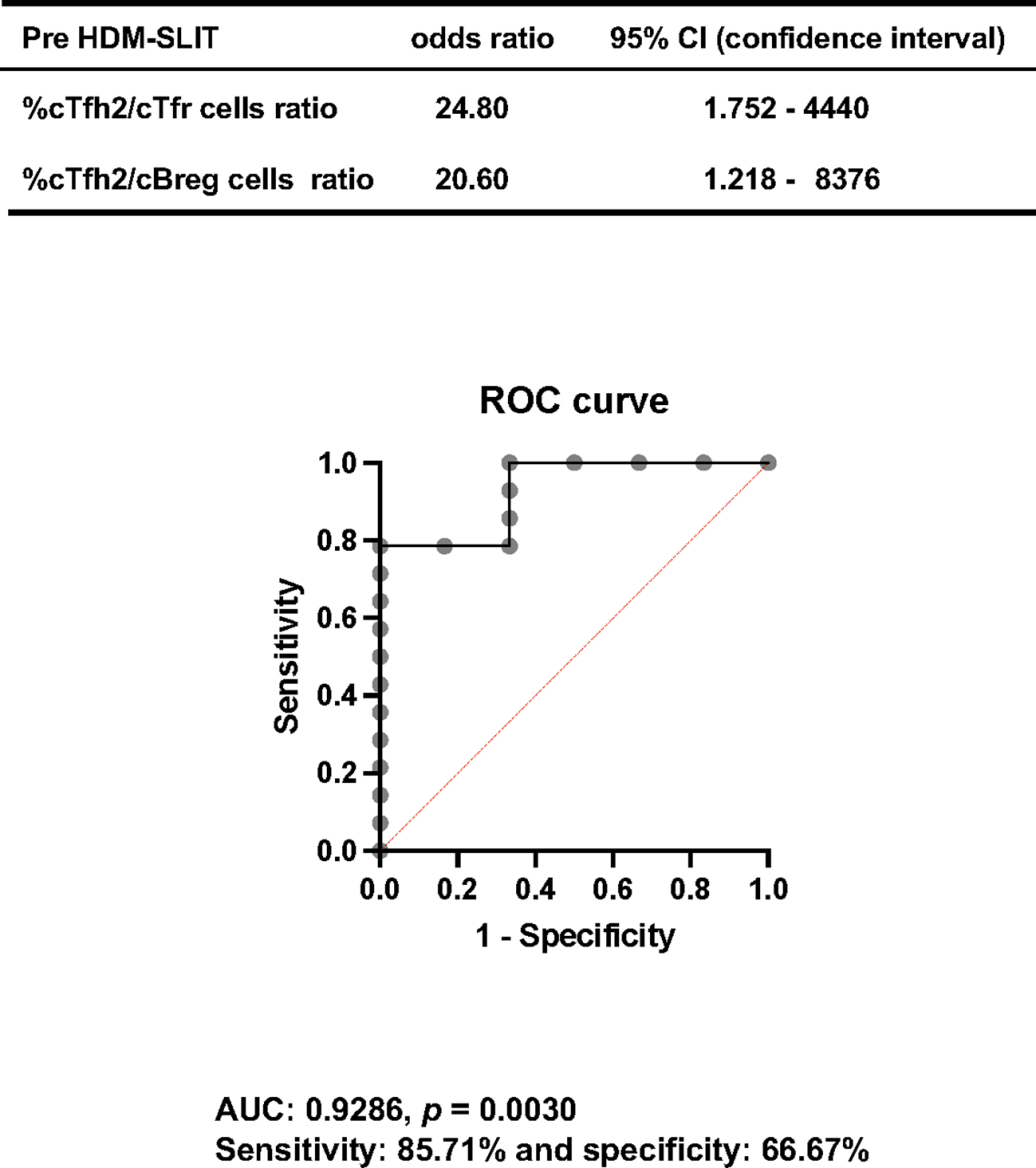
The ratio of cTfh2/cTfr cells and the ratio of cTfh2/cBreg cells at the start of HDM-SLIT showed significant odds ratios by two-way logistic analysis. The ROC curve from this analysis is shown (AUC: 0.9231, *p = 0.0038*, sensitivity: 84.62% and specificity: 66.67%).

In order to identify a predictive biomarker of a good response to HDM-SLIT, we compared the results at the start of HDM-SLIT in the responder and non-responder groups. Candidate variables in variable selection for multiple logistic regression analysis were percentages of cTfh2 cells, cTfh2/cTfh1 cells, cTfh2/cTfr cells and cTfh2/cBreg cells, which were significantly increased in the responder group (Mann–Whitney U test). We performed multi-plelogistic regression analysis with the backward variable selection method. In this analysis, the ratio of cTfh2/cTfr cells and the ratio of Tfh2/cBreg cells were selected as statistically significant predictors for the response to HDM-SLIT, and the odds ratios and 95% CI were 20.96 (95% CI: 1.260 – 8027) and 24.63 (95% CI: 1.748 – 4250), respectively. As the discrimination ability of these two variables for response to HDM-SLIT, the area under the curve (AUC) was 0.9286 (*p = 0.0030*) and the sensitivity and specificity were 85.71% and 66.67%, respectively (**Figure 4**). This logistic analysis revealed that these factors can be used as biomarkers for predicting a good response to HDM-SLIT.

## Discussion

In this study, we examined cTfh subset cells, including cTfr cells, cTreg cells and cBreg cells, and *Der-p/f* sIgs in the responder and non-responder groups. We found that the percentage of cTfh2 cells significantly decreased and the percentage of cTfh1 cells significantly increased in responders compared with those in non-responders in the first year after the start of HDM-SLIT. In the two-year course of HDM-SLIT, each level of *Der-p/f* sIgs showed characteristic findings. Remarkably, from 6 months to 12 months, the levels of *Der-p/f* sIgEs significantly decreased together with the percentage of cTfh2 cells and ratio of cTfh2/cBreg cells in responders but not in non-responders. In that period, the percentage of cTfh2 cells showed strong negative correlations with the percentages of cTreg cells and cTfr cells. We showed that the percentage of IL-4^+^ cTfh cells were significantly decreased and the percentage of IFN-γ^+^ cTfh cells were significantly increased between the start of HDM-SLIT and 24 months in patients who received HDM-SLIT. The results of multi plelogistic regression analysis revealed that the ratios of cTfh2/cTfr cells and cTfh2/cBreg cells can be used as biomarkers for predicting a good response to HDM-SLIT.

It is important that the percentage of cTfh2 cells and the levels of *Der-p/f* sIgEs were extensively reduced in the responder group from 6 months to 12 months. The percentage of cTfh2 cells showed significant negative correlations with the percentages of cTreg and cTfr cells in responders. Yao et al. reported that Tfr cells obtained from patients with AR could not suppress HDM-sIgE production in the presence of Tfh2 cells but that IgE production was decreased and the ratio of cTfr/cTfh2 cells (5) was increased by subcutaneous immunotherapy (SCIT). It has also been reported that FoxP3^(+)^ Treg cells have the capacity to suppress proliferation of T effector cells and cytokine production (22). From these reports, we speculate that the reactivity of Tfh2 cells was inhibited through the suppressor function of Treg and Tfr cells. In addition to these findings, the ratio of cTfh2/cBreg cells was significantly reduced from 6 months to 12 months in the responder group. In this study, we identified Breg cells as CD19^+^CD24^hi^CD27^+^ subpopulation of B cells which secrete IL-10 (23). We also showed the IL-10 secretion by CD19^+^CD24^hi^CD27^+^ B cells by FACS (**Supplementary Figure 3B**). We previously reported that the ratio of cTfh2/cBreg cells showed a positive correlation with total IgE titers in AR+AA patients (10) and this ratio was also correlated with serum titers of CXCL13, the counterpart of the CXCR5 receptor in AA patients (24). Anchour et al. found that human IL-10 and TGF-β-producing Breg cells controlled Tfh cell maturation and Tfr cell induction (25). From these reports, it is reasonable that the ratio of cTfh2/cBreg cells decreased from 6 months to 12 months in the responder group. We showed no correlation between cTfh2 cells and Der-p/f sIgEs in responder (unpublished data). As above described, we think that AIT progress regulatory immunity including T and B cells against allergic reaction, therefore, it is most important the balance between regulatory immunity and allergic reaction.

In this study, in addition to the decrease in the percentage of cTfh2 cells, the percentage of cTfh1 cells significantly increased in the course of HDM-SLIT. This has not been previously reported in AIT. There was no significant change in the percentage of cTfh2 or cTfh1 cells during a one-year period without HDM-SLIT. Furthermore, the percentage of IL-4^+^ cTfh cells were significantly decreased in both 4 responders and 2 non-responders and the percentage of IFN-γ^+^ cTfh cells increased except in one non-responder from the start of HDM-SLIT to 24 months. The ratio of IL-4^+^/IFN-γ^+^ in cTfh cells were lower in the 4 responders than in the 2 non-responders at 24 months. Sharif et al. reported a decrease of IL-4^+^ cTfh cells by AIT in patients with an allergy to grass pollen (4). The present study is the first study showing that IL-4^+^ cTfh cells decreased and IFN-γ^+^ cTfh cells increased in responder patients with AA+AR receiving HDM-SLIT. Some studies have shown that effective AIT results in the shift of a Th2 response toward a Th1 response (26). Guerra et al. reported that apoptosis mainly occurred in IL-4^+^ CD4 T cells but did not occur in IFN-γ^+^ CD4 cells and finally resulted in a subsequent increase of IFN-γ^+^ CD4 cells (27). Ciepiela et al. reported that Bcl-2 Th1 cells were resistant to apoptosis in patients receiving HDM-SLIT (28). Taking into account the plasticity between Tfh cells and Th cells, the cTfh1 shift that we observed might have been caused by progression of the shift from a Th2 response to a Th1 response in AIT. In recent studies, Heeringa (29) et al. and Floch (30) et al. showed elevation of antigen-specific IgG2 in SLIT responders. It is known that IgG2 is induced by IFN-γ. Further investigation is needed to elucidate the associations between Tfh1 cells and other examined cells and to determine whether Tfh1 cells are involved in HDM-specific IgG2 production in SLIT.

We evaluated immunological biomarkers of a response to HDM-SLIT based on outcomes in the two-year period. The ratios of cTfh2/cTfr and cTfh2/cBreg cells at the start of HDM-SLIT are expected to be effective biomarkers that were shown in our study to be associated with immunological findings of HDM-SLIT. These indexes need to be validated in the future.

In conclusion, both the percentage of cTfh2 cells and the levels of *Der-p/f* sIgEs significantly decreased and the percentage of cTfh1 cells significantly increased in responders in the first year after the start of HDM-SLIT. The ratio of cTfh2/cBreg cells significantly decreased from 6 months to 12 months. The percentages of cTreg and cTfr cells showed significant negative correlations with the percentage of cTfh2 cells from 6 months to 12 months. The percentage of IL-4^+^ cTfh cells significantly decreased and the percentage of IFN-γ^+^ cTfh cells increased. The results indicated the possibility that these regulatory cells greatly suppressed decreased percentage of Tfh2 cells and that the suppression is involved in the reduction of *Der-p/f* sIgEs production. The ratios of cTfh2/cTfr cells and cTfh2/cBreg cells are effective immunological biomarkers for predicting the outcome of HDM-SLIT.

## Data availability statement

I state the restrictions that apply to the dataset. Requests to access the datasets should be directed to KS, sigehara@tg8.so-net.ne.jp.

## Ethics statement

The studies involving humans were approved by The Institutional Review Board of Sapporo Medical University Hospital. The studies were conducted in accordance with the local legislation and institutional requirements. The participants provided their written informed consent to participate in this study.

## Author contributions

KS: Conceptualization, Data curation, Funding acquisition, Investigation, Methodology, Project administration, Software, Validation, Visualization, Writing – original draft, Writing – review & editing. RK: Conceptualization, Formal Analysis, Funding acquisition, Investigation, Methodology, Supervision, Writing – review & editing. II: Investigation, Supervision, Writing – review & editing. HS: Investigation, Writing – review & editing. MY: Investigation, Writing – review & editing. SK: Investigation, Writing – review & editing. KK: Investigation, Writing – review & editing. YA: Data curation, Writing – review & editing. SM: Investigation, Writing – review & editing. HN: Investigation, Writing – review & editing. EU: Investigation, Writing – review & editing. KY: Funding acquisition, Writing – review & editing. KT: Supervision, Writing – review & editing. HC: Supervision, Writing – review & editing. HO: Methodology, Software, Supervision, Writing – review & editing. SI: Conceptualization, Project administration, Supervision, Writing – review & editing.

## Supplementary material

Supplementary Figure 1 ACQ: ACQ scores showed significant interaction, response and time course. GINA treatment step score: GINA medication stage score also showed a significant interaction between the two groups and decreased significantly in the responder group during the two-year period. cSMS: cSMS showed a significant interaction between two groups and cSMS significantly decreased in responders compared with those at the start of HDM-SLIT during the two-year period. (*^★^p < 0.05 ^★ ★^p < 0.01*, *^★★★^p < 0.001*, *^★★★★^p < 0.001 compared with pre-SLIT*).

Supplementary Figure 2 FACS procedures. **(A)** cTfh cells and their subset cells and PD-1 and ICOS expression. **(B)** cTreg and cTfr cells. **(C)** Expression of IL-4^+^ and IFN-γ^+^ in cTfh cells.

Supplementary Figure 3 FACS procedures. **(A)** CD3^-^CD19^+^CD24^hi^CD27^+^ cells (Breg cells). **(B)** IL-10 secretory CD3^-^CD19^+^CD24^hi^CD27^+^ cells (Breg cells).

Supplementary Figure 4 The percentage of cTfh17 cells, cTreg cells and cTfr cells. And the absolute numbers of cTfh subsets cells, cTreg cells and cTfr cells. The percentage of cTreg cells significantly increased at 6 months in responders. The absolute numbers of cTfh1 cells showed significant interaction and a significant increased at 24 months in responders. (*^★^ p < 0.05, ^★ ★^ p < 0.01 compared with pre-SLIT*).

Supplementary Figure 5 The percentage and the absolute numbers of cB(total) cells, c-memory B cells, c-plasmablast, and cBreg cells. The percentage of c-memory B cells in non-responders significantly increased at 6 months (***^‡‡‡^*** *p < 0.001*) and 18 months (***^‡^*** *p <0.05*) compared with those in responders. The percentage of cBreg cells in responders significantly decreased from 3 months to 6 months (*^★^ p < 0.05*).

Supplementary Table 1 Characteristics of patients.

Supplementary Table 2 Monoclonal antibodies used in this study.
