# Supplementary figures and images for: Circulating T follicular helper 2 cells, T follicular regulatory cells and regulatory B cells are effective biomarkers for predicting the response to house dust mite sublingual immunotherapy in patients with allergic respiratory diseases
Source: Front Immunol. 2023 Nov 24;14:1284205. doi: 10.3389/fimmu.2023.1284205 (PMC10726700; doi:10.3389/fimmu.2023.1284205)

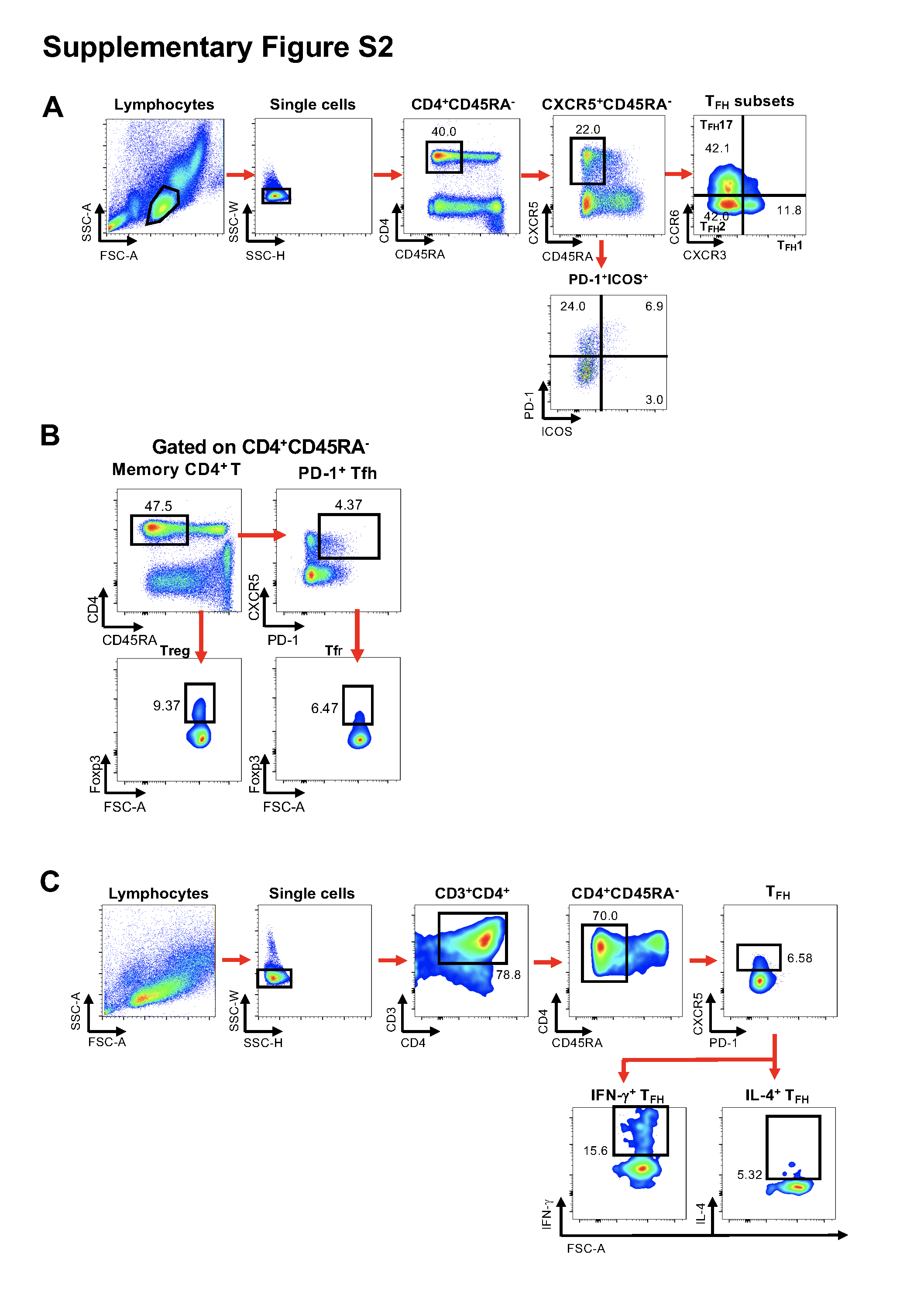

Supplement: Supplementary Figure 2 — FACS procedures. (A) cTfh cells and their subset cells and PD-1 and ICOS expression. (B) cTreg and cTfr cells. (C) Expression of IL-4+ and IFN-γ+ in cTfh cells. [file Image_2.tiff]

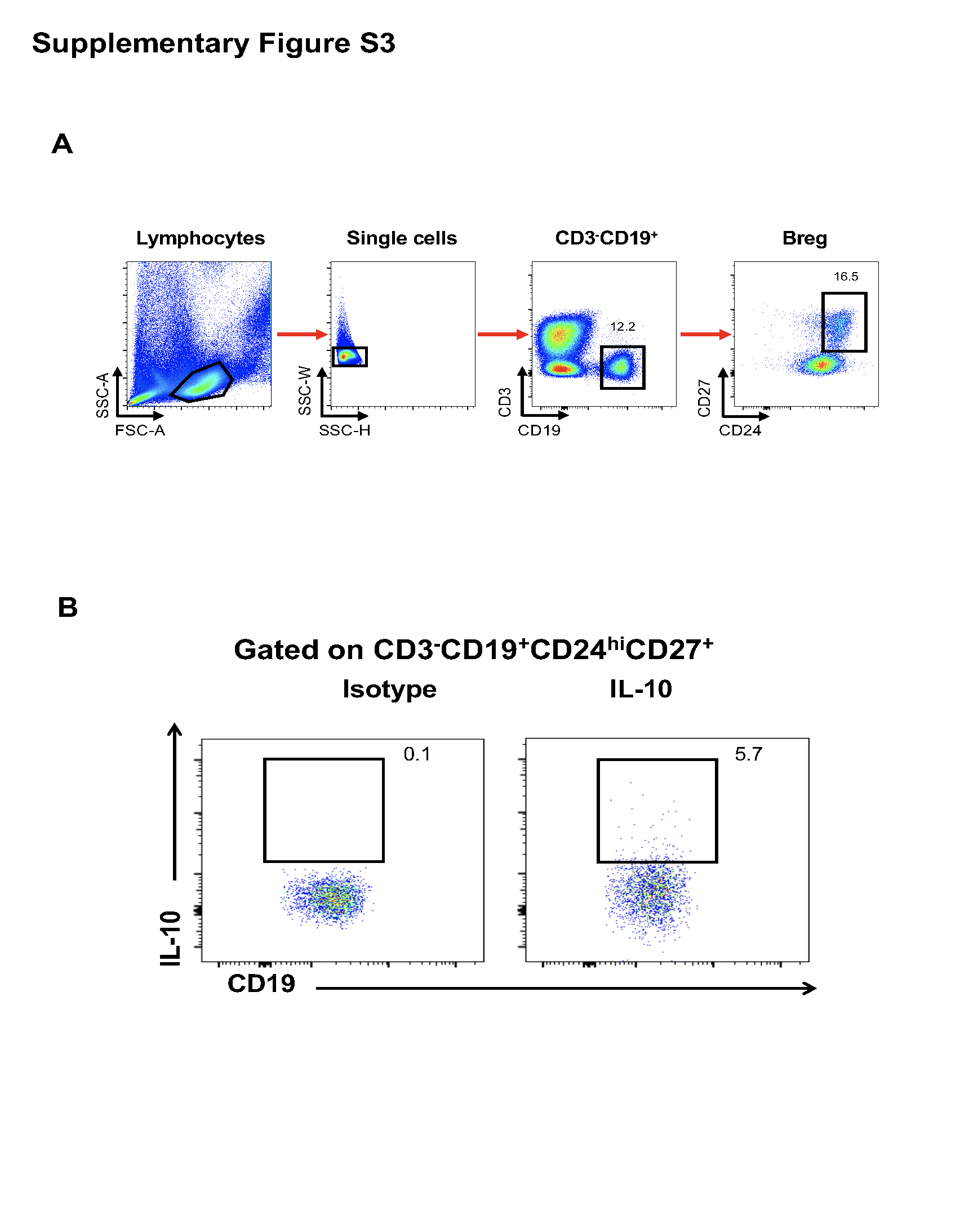

Supplement: Supplementary Figure 3 — FACS procedures. (A) CD3-CD19+CD24hiCD27+ cells (Breg cells). (B) IL-10 secretory CD3-CD19+CD24hiCD27+ cells (Breg cells). [file Image_3.tiff]
